# Identification of Short Hairpin RNA Targeting Foot-And-Mouth Disease Virus with Transgenic Bovine Fetal Epithelium Cells

**DOI:** 10.1371/journal.pone.0042356

**Published:** 2012-08-08

**Authors:** Hongmei Wang, Jianming Wu, Xiao Liu, Hongbin He, Fangrong Ding, Hongjun Yang, Lei Cheng, Wenhao Liu, Jifeng Zhong, Yunping Dai, Guangpeng Li, Chengqiang He, Li Yu, Jianbin Li

**Affiliations:** 1 Dairy Cattle Research Center, Shandong Academy of Agricultural Sciences, Jinan, People's Republic of China; 2 State Key Laboratory for Agrobiotechnology, China Agricultural University, Beijing, People's Republic of China; 3 College of Life Science, Inner Mongolia University, Huhehaote, People's Republic of China; 4 College of Life Science, Shandong Normal University, Jinan, People's Republic of China; 5 Division of Livestock Infectious Diseases, State Key Laboratory of Veterinary Biotechnology, Harbin Veterinary Research Institute, Harbin, People's Republic of China; University of Kansas Medical Center, United States of America

## Abstract

**Background:**

Although it is known that RNA interference (RNAi) targeting viral genes protects experimental animals, such as mice, from the challenge of Foot-and-mouth disease virus (FMDV), it has not been previously investigated whether shRNAs targeting FMDV in transgenic dairy cattle or primary transgenic bovine epithelium cells will confer resistance against FMDV challenge.

**Principal Finding:**

Here we constructed three recombinant lentiviral vectors containing shRNA against VP2 (RNAi-VP2), VP3 (RNAi-VP3), or VP4 (RNAi-VP4) of FMDV, and found that all of them strongly suppressed the transient expression of a FLAG-tagged viral gene fusion protein in 293T cells. In BHK-21 cells, RNAi-VP4 was found to be more potent in inhibition of viral replication than the others with over 98% inhibition of viral replication. Therefore, recombinant lentiviral vector RNAi-VP4 was transfected into bovine fetal fibroblast cells to generate transgenic nuclear donor cells. With subsequent somatic cell cloning, we generated forty transgenic blastocysts, and then transferred them to 20 synchronized recipient cows. Three transgenic bovine fetuses were obtained after pregnant period of 4 months, and integration into chromosome in cloned fetuses was confirmed by Southern hybridization. The primary tongue epithelium cells of transgenic fetuses were isolated and inoculated with 100 TCID_50_ of FMDV, and it was observed that shRNA significantly suppressed viral RNA synthesis and inhibited over 91% of viral replication after inoculation of FMDV for 48 h.

**Conclusion:**

RNAi-VP4 targeting viral VP4 gene appears to prevent primary epithelium cells of transgenic bovine fetus from FMDV infection, and it could be a candidate shRNA used for cultivation of transgenic cattle against FMDV.

## Introduction

Foot-and-mouth disease (FMD) is a severe, clinically acute, vesicular disease of cloven-hoofed animals, including cattle, swine, and sheep, as well as more than 70 species of wild animals, its outbreaks have occurred in every livestock-containing region of the world with the exception of New Zealand [1]. Although FMD does not result in high mortality in adult animals, the disease has debilitating effects, including weight loss, decrease in milk production, and loss of draught power, resulting in a loss in productivity for a considerable time. However, mortality can be high in young animals, where the virus can affect the heart. In addition, cattle, sheep, and goats can become carriers, and cattle can harbor virus for up to 2 to 3 years [2]. The etiological agent of FMD is foot-and-mouth disease virus (FMDV), which is the type species of the *Aphthovirus* genus of the *Picornaviridae* family. The presence of seven serotypes and multiple subtypes and variants has added to the difficulty of laboratory diagnosis and control of FMD. The rise of new variants is inevitably caused by continued circulation of the virus in the field and the quasispecies nature of the RNA genome [3], [4]. Therefore, FMD is on the A list of infectious diseases of animals of the Office International des Epizooties (OIE) and has been recognized as the most important constraint to international trade in animals and animal products [5].

The introduction of the killed FMD vaccine has been extremely successful in reducing the number of disease outbreaks in many parts of the world where the disease is enzootic. However, there are a number of concerns and limitations with its use in emergency control programs [1]. For example, the antigenic variation within FMDV makes viruses easily escape from the host immune systems. Furthermore, vaccination may induce immunologic pressure within the population that could result in the emergence of a new variant [1]. In addition, vaccines are serotype specific, there are no cross protective reaction among different serotypes. Moreover, the signs of FMD can appear as early as 2 days post exposure, however, current vaccines do not induce a protective response until 7 days post vaccination. Thus, early protection is required in the event of an FMD outbreak in a disease-free country to prevent virus amplification and disease spread [6]. Targeting virus using RNA interference (RNAi) is one of the possible alternative strategies for FMDV control because it is a rapid and effective antiviral approach, which can be used as an emergency for suspected cases, including persistently infected or susceptible animals [7].

Short hairpin RNA (shRNA) can be designed to hybridize a particular viral mRNA to promote its degradation, thus serving as an effective antiviral approach to protect either plants [8] or animal species [9], [10] from viruses. This approach is a highly specific tool to down-regulate gene expression [11] and has been extensively utilized to inhibit FMDV *in vitro* and/or *in vivo* [6], [12], [13], [14], [15], [16], [17], [18]. Furthermore, transgenic mice expressing FMDV targeting shRNA was much more resistant to viral infection, as evidenced by minor abnormal pathology, as compared to the control mice after challenge with FMDV [19]. However, little has been published regarding function of shRNA targeting FMDV for the prevention and control of FMD in transgenic cattle.

In fact, transgenic farm animals have been developed and showed disease resistance. For example, expression of lysostaphin in mammary gland combated mastitis and protected transgenic cattle from mammary gland challenges by Staphylococcus aureus [20], [21], [22]. Therefore, it is possible to address disease problems in agriculturally important species using transgenic technology [21], and genetic engineering can provide a viable tool for enhancing resistance to disease and improve the well-being of livestock.

In this study, RNAi targeting VP2, VP3, and VP4, respectively, were constructed into Lenti-virus vectors. All shRNAs could silence viral gene in 293 cells and inhibit replication of FMDV in BHK-21 cells. Lenti-RNAi-VP4 vector was transfected into bovine fetal fibroblast cells, and transgenic stable cells were screened and selected as nuclear donor cells for subsequent somatic cell cloning. Three transgenic bovine fetuses expressing this shRNA were obtained, and their primary tongue epithelium cells were used to evaluate shRNA targeting effect against FMDV.

## Results

### shRNA recombinant lentivirus vectors inhibiting transient expression of viral genes in 293T cells

**Figure 1:**
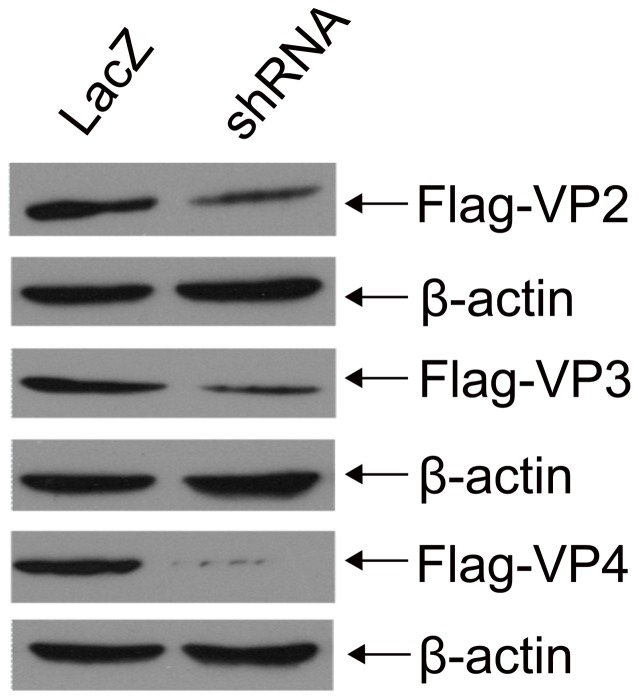
shRNAs silencing transient expression of viral genes in 293T cells. Cell lysates of 293T cells co-transfected with shRNA recombinant lentivirus vectors and either pcDNA3-Flag-VP2, pcDNA3-Flag-VP3, or pcDNA3-Flag-VP4 were separated by SDS-PAGE, and then analyzed by Western blotting using antibodies against Flag and β-actin. Three experiments were independently repeated.

We generated three lentiviral shRNAs targeting VP2 (Lenti-RNAi-VP2), VP3 (Lenti-RNAi-VP3), and VP4 (Lenti-RNAi-VP4), respectively, and co-transfected each individual construct with pcDNA3-FLAG-VP2, pcDNA3-FLAG-VP3, or pcDNA3-FLAG-VP4, respectively, into 293T cells. Expression of FLAG-VP2, FLAG-VP3, or FLAG-VP4 was determined by Western Blotting. As shown in Figure 1, shRNAs against viral genes significantly inhibited transient expression of FLAG-VP2, FLAG-VP3, or FLAG-VP4 as compared to the LacZ-control shRNA. RNAi-VP4 almost completely eliminated expression of VP4. These results strongly suggested a direct role of shRNAs in specifically blocking transient expression of viral proteins in 293T cells.

### shRNA silencing of viral genes and inhibition of FMDV replication in BHK-21 cells

**Figure 2:**
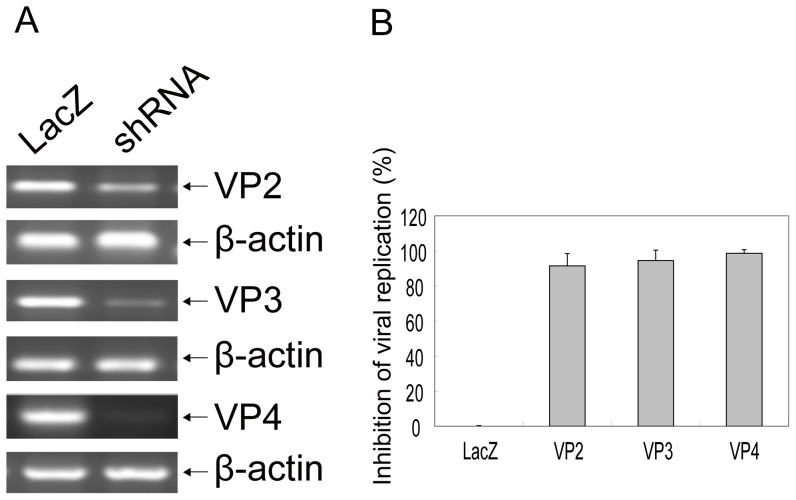
shRNAs targeting viral gene and inhibiting replication of FMDV in BHK-21 cells. BHK-21 cells stable expressing shRNA were cultured in 12-well plates in four replicates, each well inoculated with 100 TCID_50_ of the FMDV ASIA1/YS/CHA/05 strain. 48 hrs after FMDV inoculation, the cells and virus were collected, and mRNA expression of viral genes was determined by RT-PCR, β-actin as control (A), and FMDV viral replication was determined by TCID_50_ (B). The results from three independent experiments in quadruplicate were presented as 1 minus a percentage of average TCID_50_ in treated cells to that in LacZ shRNA expressed control cells± one standard deviation.

We next determined whether shRNAs could block natural expression of viral genes and inhibit FMDV replication. The BHK-21 cell lines stably expressing shRNAs were sorted by fluorescence-activated cell sorter (FACS) analysis based on eGFP co-expression in the viral vector. The stable clones were inoculated with 100 TCID_50_ of the FMDV strain, viral gene mRNA expression and viral replications were determined. As shown in Figure 2A, expression of viral gene mRNAs was strongly inhibited by either RNAi-VP2, RNAi-VP3, or RNAi-VP4 as compared to control shRNA (LacZ). β-actin mRNA expression by contrast was not altered by either shRNA. By TCID_50_ Assay, RNAi-VP2 inhibited over 91% of viral replication as compared to LacZ control, while RNAi-VP3 showed more than 94% inhibition of viral replication. RNAi-VP4 inhibited over 98% of viral replication (Figure 2B). Thus, shRNAs specifically silenced viral gene mRNA and reduced viral replication *in vitro*.

### Establishment of transgenic bovine fetal fibroblast cells expressing shRNA against VP4 gene of FMDV

**Figure 3:**
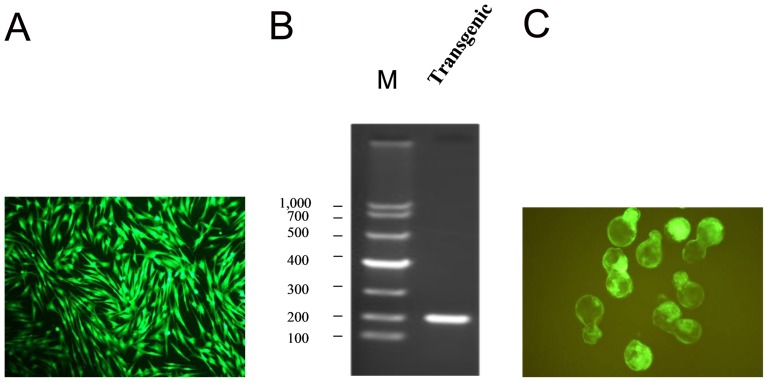
Development of reconstructed transgenic embryos. shRNA recombinant lentivirus vector RNAi-VP4 was transfected into bovine fetal fibroblast cells using Lipofectamine 2000, transgenic bovine fetal fibroblast cells were screened by FACS, all of them expressed eGFP (A), and confirmed by PCR with primer pair LT1/LT2 (B) and DNA sequencing. shRNA transgenic cells were transferred into enucleated oocyte cytoplasts, and transgenic blastocysts were screened based on their development and eGFP expression (C).

We then transfected Lenti-RNAi-VP4 targeting FMDV into bovine fetal fibroblast cells. All transgenic bovine fetal fibroblast cells expressed eGFP after screened by FACS based on shRNA recombinant H1 lentivirus encoding eGFP (Figure 3A), and were confirmed by PCR (Figure 3B) and DNA sequencing.

### Development of reconstructed embryos

We generated a total of 215 reconstructed oocytes after transgenic cells expressing RNAi-VP4 were transferred into enucleated oocyte cytoplasts. The fusion, cleavage and blastocyst development rates were 81.8% (176/215), 83.5% (147/176), and 30.6% (45/147), respectively. Forty blastocysts were screened based on their development and eGFP expression (Figure 3C), and then transferred to 20 synchronized recipient cows. Pregnancy rates at 60 days were 25% (5/20); three 4-month-old fetuses were obtained after pregnancy of 4 months.

### Lenti-RNAi-VP4 integration into chromosome of cloned bovine fetuses

**Figure 4:**
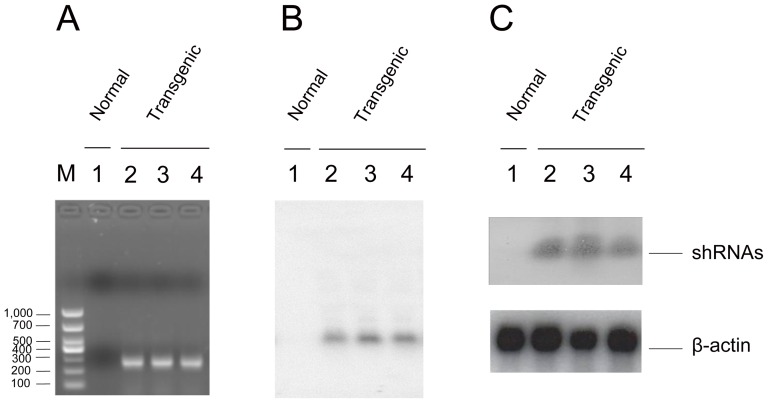
shRNA integration into chromosome of cloned fetuses and expression in primary transgenic bovine tongue epithelium cells. The genomic DNA from the ear tissue of one normal (lane 1) and three transgenic (lane 2, 3, and 4) fetuses were extracted, and used as the template for PCR amplification, there was positive band in cloned fetuses, but not in normal fetus (A), shRNA transgenic fragment was confirmed by DNA sequencing. The genomic DNAs then were digested into smaller fragments with excess amounts of *Xho*I and *Apa*I for Southern Blotting, and were hybridized with probe (B). The expression of shRNA in primary transgenic bovine tongue epithelium cells was determined by Northern analyses (C), β-actin as a quantitative controls. Three experiments were independently repeated.

We next determined if Lenti-RNAi-VP4 was indeed integrated into chromosome of cloned bovine fetuses after the detection of expected shRNA-VP4, fragments by PCR and confirmed by DNA sequencing in cloned fetuses, but not in normal fetuses (Figure 4A). Both Southern Blotting (Figure 4B) and Northern (Figure 4C) analyses revealed integrated genomic band and RNAi-VP4 expression, respectively in transgenic, but not in normal fetuses. Thus, the shRNA targeting VP4 was indeed integrated into chromosome.

### Targeted viral RNA degradation by shRNAs in primary transgenic bovine tongue epithelium cells

**Figure 5:**
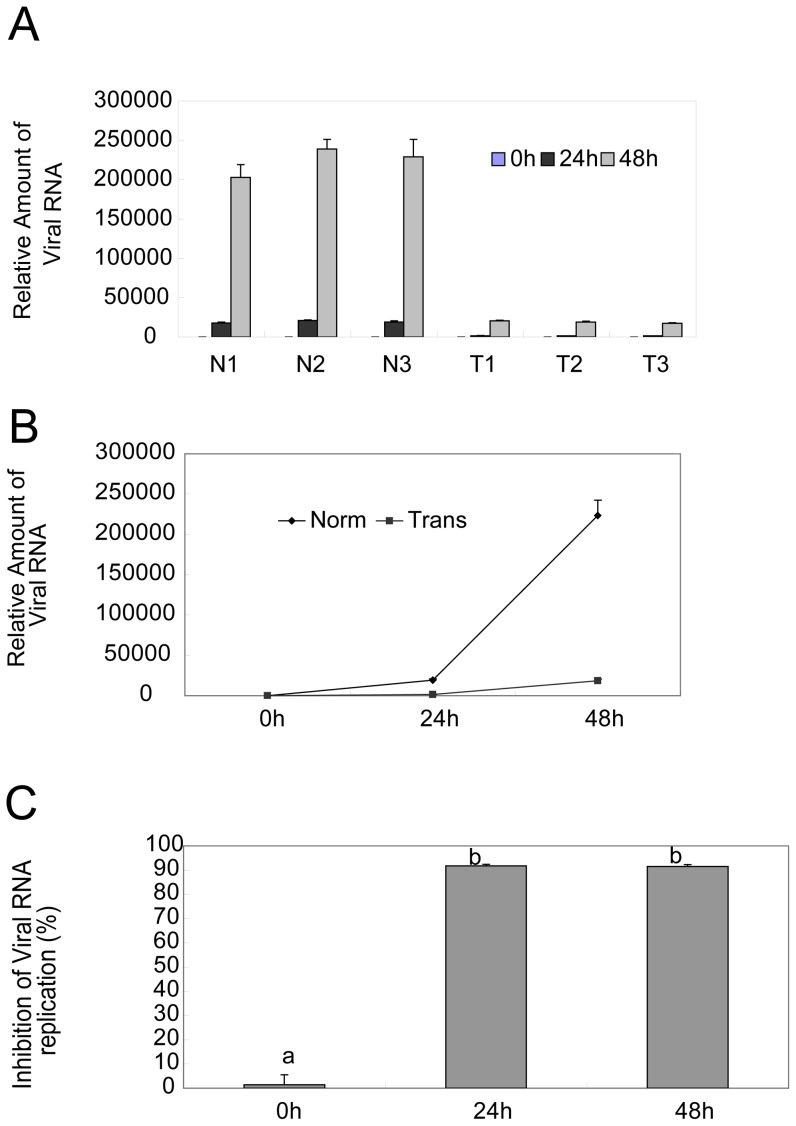
shRNAs degradation of viral RNA in primary transgenic bovine tongue epithelium cells. The primary bovine tongue cells were isolated from three normal (N1, N2, N3) or three transgenic (T1, T2, T3), respectively, and inoculated with 100 TCID_50_, viral samples were taken at 0 h, 24 h and 48 h, respectively, and viral RNA was determined with real-time RT-PCR, the relative amount viral RNA in three normal (N1, N2, N3) or three transgenic (T1, T2, T3) primary bovine tongue cells from three independent experiments was computed using ABI Prism 7000 SDS Software (Applied Biosystems) (A), the average amount viral RNA was compared between normal (Norm) and transgenic (Trans) groups (B), and its inhibition by shRNA in transgenic cells was evaluated as compared to normal controls (C). The standard used was 10-fold dilutions of 10^7^ TCID_50_ of virus ml^−1^. Duncan's multiple range test by SAS 8.0 system, means with the same letter are not significantly different (P>0.01).

To directly show viral RNA degradation by shRNAs, we inoculated the tongue epithelium cells with the virus at a titer of 100 TCID_50_, and collected the viral samples at indicated time points, followed by viral RNA determination by real-time RT-PCR. As shown in Figure 5A (a representive result of three independent experiments), there was significant difference in relative amounts of viral RNA between cells from three transgenic (T1, T2, and T3) and normal fetuses (N1, N2, and N3). The average viral RNA in three transgenic cells were 1576 and 18974 at 24 h and 48 h, respectively, as compared to 19275 and 223643 in normal cells (Figure 5B). So shRNAs expressed in transgenic cells inhibited over 91% of viral RNA replication as compared to normal control cells (Figure 5C). Thus, shRNA expression in transgenic cells significantly degraded viral RNA.

### shRNAs protection of primary transgenic bovine tongue epithelium cells from FMDV infection

**Figure 6:**
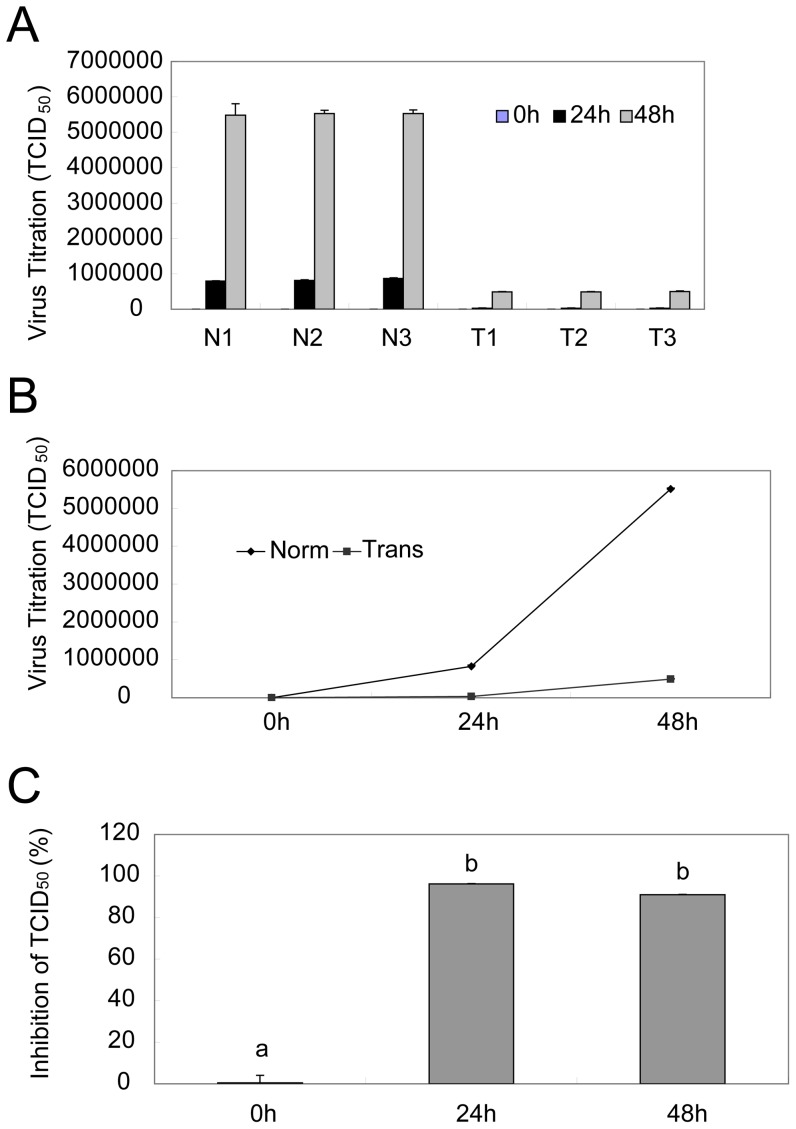
shRNAs protection of primary transgenic bovine tongue epithelium cells from FMDV infection. The primary bovine tongue cells were isolated from three normal or three transgenic fetuses, respectively, cultured in 96-well plates and inoculated with 100 TCID_50_ of the FMDV strain each well. Virus samples were collected at indicated time points, and virus titer was then determined. The viral titers in three normal (N1, N2, N3) or three transgenic (T1, T2, T3) cells from three independent experiments were presented as an average number of TCID_50_ ± one standard deviation at different time (A), the average viral titers was compared between normal (Norm) and transgenic groups (Trans) (B), and viral inhibition by shRNA in transgenic cells was calculated as 1 minus percentage of average number of TCID_50_ of transgenic cells to that of normal controls (C). Duncan's multiple range test by SAS 8.0 system, means with the same letter are not significantly different (P>0.01).

Finally, we determined if shRNAs protects cells from virus infection. To this end, we determined the viral replications after inoculation of 100 TCID_50_ of FMDV. While there is no difference in viral titer among cells expressing three transgenic shRNA (T1, T2, and T3), or among corresponding normal controls (N1, N2, and N3), significance exists when transgenic cells were compared to the normal controls (Figure 6A). The average viral titers in three normal primary bovine fetus tongue epithelium cells were about 825679 and 5517964 TCID_50_ at 24 and 48 h, respectively, whereas the average titers in transgenic cells were 31258 and 492618 TCID_50_, respectively (Figure 6B). Thus, the shRNAs expression in transgenic cells caused about 96.2% or 91.1% inhibition of viral replication (Figure 6C), respectively. We concluded from this study that, expression of shRNAs in primary transgenic bovine tongue epithelium cells significantly reduced viral replication.

## Discussion

It was well-established that RNAi targeting viral genes could protect experimental animals from FMDV challenge, and the protection occurs rapidly and was specific. For example, neck subcutaneous injection of a multiple RNAi plasmid targeting VP1 gene rendered the suckling mice much less susceptible to FMDV serotype O and Asia I [12], [14]. Likewise, shRNA targeting 3D gene protected guinea pigs [15] and swine [13] from challenge virus. Moreover, an attenuated Salmonella choleraesuis-mediated siRNA targeting 3D gene protected guinea pigs and swine against FMDV [23], respectively. Although RNAi has been used as an effective antiviral strategy due to its specific silencing of viral gene expression, thus effectively controlling the severity of FMD infection and spread [13], [15], the pivotal issues of RNAi based anti-viral strategy are the delivery and stability of the RNAi reagents with delivery being the major hindence [24]. At the present time, there is no clinical application of RNAi targeting approach in farm. Given the fact that transgenic mice expressing FMDV targeting shRNA became much more resistant to the challenge of FMDV serotype Asia 1 [19], transgenic cloning may be a useful tool for RNAi anti-viral strategy. Currently, no studies of transgenic shRNA function in cloven-hoofed animals have been reported.

FMDV consists of a single-stranded RNA genome of approximately 8.5 kb, the RNA is translated as a single long open reading frame into a long poly-peptide, which is undergone a series of post-translational proteolytic cleavages to generate different viral proteins, so RNAi may be screened based upon its ability to degrade the viral RNA. In fact, VP1, VP4, 3D, 2B, 5′NCR, VPg, or 3′NCR have been previously selected as RNAi target genes [6], [12], [13], [14], [15], [16], [17], [18]. If there are conserved regions of viral genes among different serotypes of FMDV, the viral genes could be screen as RNAi targets, so we choose RNAi-VP2, RNAi-VP3, RNAi-VP4 as target site in this study. We observed that shRNA targeting VP2, VP3 or VP4 gene of FMDV could silence expression of viral genes in 293T cells (Figure 1), and inhibit over 91%, 94% and 98%, respectively, of viral replication in BHK-21 cells (Figure 2B). We achieved a very high level of inhibition of virus replication, likely due to selection of stable clones in which most cells expressed targeting shRNAs. The transient transfection of RNAi expressing plasmids/siRNA into BHK-21 cells was a common delivery method [6], [12], [14], [16], [17], [18], with limitation of transfection efficiency, shRNA/siRNA was not expressed in most cells, resulting in an reduced inhibition of viral replication. In our study, stable BHK-21 cell lines expressing targeting shRNAs were selected via FACS sorting for eGFP, which is co-expressed in H1 Lenti-virus vector. Thus, to evaluate efficiency of any given candidate RNAi, the stable clones should be established in order to achieve a high level of anti-virus activity.

In this study, we transfected RNAi-VP4 into bovine fetal fibroblasts cells, followed by transfering the transgenic cells into enucleated oocyte cytoplasts, selection of reconstructed embryos were selected based on their expression of eGFP (Figure 3C) and finally transferring the reconstructed embryos to synchronized recipient cows. Since the major focus of our study is to evaluate the efficiency of FMDV shRNA targeting after transgenic delivery, we used 4-month-old fetuses, insteading of adult animals, for the sake of saving time and money. We confirmed shRNA integration into chromosome of cloned fetuses by Southern Blotting and the expression of shRNA by Northern. Since four-month-old transgenic fetuses could not survive in vitro, for FMDV challenge assay, we used primary tongue epithelium cells established from small pieces of the mucosa collected from the tip of bovine tongue. Since the targeting sequence of RNAi-VP4 was conserved among O, A, and Aisa1 serotypes of FMDV, and ASIA1/YS/CHA/05 strain is able to grow well in BHK-21 cells and in primary tongue epithelium cells, we used ASIA1/YS/CHA/05 strain as challenge virus in this study. We found that shRNA expressed in transgenic fetuses could significantly degrade viral RNA after inoculation of FMDV at a titer of 100 TCID_50_ (Figure 5), and inhibited viral replication (Figure 6). Thus, primary transgenic bovine fetus tongue epithelium cells became much more resistant to FMDV challenge.

The most important threat caused by FMDV is the high speed of viral replication, short incubation time, and high contagiousness. Thus, although protective immune responses against FMDV can be efficacious, the rapidity of virus replication and spread can outpace the development of immune defenses and overrun the immune system [25]. Our observation that shRNA inhibited over 91% of viral replication at 48 h after challenge (Figure 6C) suggest that RNAi-based virus targeting is useful for transgenic cows to get more time to develop immune defense. Needless to say, whether transgenic cows indeed become resistant to FMDV infection will wait for the future study using adult transgenic cows upon FMDV challenge. In fact, we have so far obtained on six-month old male transgenic dairy cattle (data not shown). The investigations in this area will help to improve the design of transgenic genes and the development of RNAi-based strategies against FMD.

Due to their high degree of sequence specificity, shRNAs become ineffective in the presence of escape mutations within and outside the targeted regions [26], and effective silencing of a single viral gene does not always translate into antiviral effect due to genetic compensation or redundancy [27], [28], [29]. Furthermore, variations within multiple regions of the quasispecies of FMDV were retrospectively revealed by sequencing of FMDV genes, strategies to inhibit RNA virus multiplication based on the use of siRNAs have to consider the high genetic polymorphism exhibited by this group of virus. Thus, it may be important to use multiplex shRNAs [30] if RNAi is to be developed for therapeutic use. In this study, we used shRNAs targeting of viral genes VP2, VP3, and VP4, and observed a significant inhibition of FMDV. Combination of these shRNAs may be necessary to avoid the evolution of escape variants.

In conclusion, we obtained three transgenic fetuses expressing RNAi-VP4 against FMDV. Study using primary tongue epithelium cells derived from these fetuses reveal that RNAi-VP4 degraded viral RNA and inhibited viral replication. This shRNA merits further investigation for cultivation of transgenic cattle against FMDV.

## Materials and Methods

Unless otherwise noted, all reagents used were obtained from Sigma Chemical Co. (St. Louis, MO, USA). All procedures were approved by the Shandong Academy of Agricultural Sciences Animal Care and Use Committee.

### Cells

BHK-21 and 293T cells were obtained from the American Type Culture Collection and grown in Dulbecco's modified Eagle's medium (DMEM) supplemented with 10% fetal bovine serum (FBS). Primary bovine tongue epithelium cells were obtained as follows, small pieces from the mucosa of the tip of a bovine tongue were used as explants to initiate a cell culture with Iscove's Dulbecco modified Eagle medium (DMEM) and Ham's F12 medium at 1∶1 supplemented with 10% fetal calf serum, and an outgrowth of polymorphic cells but mostly epithelium-like cell types was observed.

### Titration of FMDV

BHK-21 cells or primary bovine tongue cells were passaged in 96-well plates with Iscove's Dulbecco modified Eagle medium (DMEM) and Ham's F12 medium at 1∶1 supplemented with 10% fetal calf serum (FCS), and infected with 10-fold dilutions of FMDV ASIA1/YS/CHA/05 strain in four replicates per dilution when they developed a confluent monolayer. After 48 hours, viral cytopathic effect (CPE) was monitored and 50% tissue infective dose (TCID_50_) of virus was calculated using the Reed-Muench method.

### Construction of shRNA recombinant lentivirus vectors and viral genes recombinant pcDNA3

Lentivirus-based shRNA vector were constructed as described previously [31]. The sequences of these of shRNA oligonucleotides are RNAi-VP2-P1: 5′- CTC TGC TTG AAG ACC GCA TTT CAA GAG AAT GCG GTC TTC AAG CAG AGT TTT TTG T -3′ and RNAi-VP2-P2: 5′- CTA GAC AAA AAA CTC TGC TTG AAG ACC GCA TTC TCT TGA AAT GCG GTC TTC AAG CAG AG -3′; RNAi-VP3-P1: 5′- GTA CCA TTT GTG AAG ACG GTT CAA GAG ACC GTC TTC ACA AAT GGT ACT TTT TTG T -3′ and RNAi-VP3-P2: 5′- CTA GAC AAA AAA GTA CCA TTT GTG AAG ACG GTC TCT TGA ACC GTC TTC ACA AAT GGT AC -3′; RNAi-VP4-P1: 5′- CCA GTC AGG CAA CAC TGG ATT CAA GAG ATC CAG TGT TGC CTG ACT GGT TTT TTG T -3′ and RNAi-VP4-P2: 5′- CTA GAC AAA AAA CCA GTC AGG CAA CAC TGG ATC TCT TGA ATC CAG TGT TGC CTG ACT GG -3′; The control shRNA sequences are LacZ-P1: 5′- CAG TTG CGC AGC CTG AAT GTT CAA GAG ACA TTC AGG CTG CGC AAC TGT TTT TTG T-3′ and LacZ-P2: 5′- CTA GAC AAA AAA CAG TTG CGC AGC CTG AAT GTC TCT TGA ACA TTC AGG CTG CGC AAC TG-3′. These oliognucleotides were annealed to each other and ligated into H1 lentivirus vector, followed by DNA sequence confirmation. The sequence of LacZ shRNA was not homologous to those of viral genes, so shRNA of LacZ recombinant lentivirus was negative shRNA control. The PCR primer pair was LT1: 5′- TGTCGCTATGTGTTCTGGGA-3′ and LT2: 5′- GGT ACA GTG CAG GGG AAA GA-3′.

Viral genes recombinant pcDNA3 plasmids were constructed. The PCR primer pair for pcDNA3-Flag-VP2, pcDNA3-Flag-VP3, and pcDNA3-Flag-VP4 were used as follows, VP2-P1: 5′- GGG GTA CC G CCA CCA TGG ACT ACA AGG ACG ACG ATG ACA CTG ACA AGA AAA CGG AGG AGA-3′ and VP2-P2: 5′- GCT CTA GAT TAC TCT TTC GAG GGC AGT TCT -3′; VP3-P1: 5′- GGG GTA CC G CCA CCA TGG ACT ACA AGG ACG ACG ATG ACA CTG GGA TAG TTC CTG TGG CGT GTG T -3′ and VP3-P2: 5′- GCT CTA GAT TAC TGT TGG CGG GCA TCC A -3′; VP4-P1: 5′- GGG GTA CC G CCA CCA TGG ACT ACA AGG ACG ACG ATG ACA CTG GAG CCG GGC AAT CCA GT-3′ and VP4-P2: 5′- GCT CTA GAT TAA GCC AAA AGA GCA CCA AAC A -3′; PCR fragments were digested with Kpn I and Xba I, and subcloned into previously digested pcDNA3, followed by DNA sequence confirmation.

### Co-transfection of lentiviral shRNAs and FLAG-tagged viral genes in 293T cells

5 µg of pcDNA3-FLAG-viral genes were co-transfected into 293T cells with 5 µg of recombinant relevant shRNA lentivirus vectors, using Lipofectamine 2000, according to manufacturer's instruction (Invitrogen), respectively. After 48 h, transient expression of the FLAG-viral gene fusion proteins were determined by Western Blot, performed as described previously [32]. In brief, 293T cells were lysed using Lysis Buffer (20 mM Tris-HCl, pH 8.0, 150 mM NaCl, 1% Triton, 5 mM EGTA, 5 mM EDTA, 1 mM NaF, 1 mM Na3VO4, freshly added proteinase inhibitor tablet) and supernatants were collected by centrifugation. Proteins were separated on polyacrylamide gels in the presence of SDS and electrophoretically transferred onto nitrocellulose membrane. The membranes were blocked with 5% Blotto in TBS-T (20 mM Tris–HCl, pH 7.4, 150 mM NaCl, 0.1% Tween-20) for 1 hr at room temperature and probed with various antibodies against FLAG and β-actin (Sigma). Specific proteins were visualized by ECL (Amersham Biosciences) detection. Viral gene expression was quantified relative to the LacZ shRNA control.

### Establishment of transgenic BHK-21 cells with shRNA and FMDV infection

5 µg of recombinant shRNA lentivirus vectors was transfected into BHK-21 cells using Lipofectamine 2000, according to manufacturer's instruction (Invitrogen), respectively. The cells were subcultured after 48 h, and stable cell lines with shRNAs were screened by FACS analysis based on shRNA recombinant H1 lentivirus encoding eGFP. The stable cell lines with RNAi-VP2, RNAi-VP3, RNAi-VP4 or LacZ shRNA were cultured in 12-well plates in four replicates, and each well inoculated with 100 TCID_50_ of the FMDV ASIA1/YS/CHA/05 strain. After 48 hours, CPE of each well was measured. Cells and supernatant were collected for assessment of shRNA gene silencing efficiency and FMDV titer, respectively. The mRNA expression of viral genes in cells transfected with recombinant shRNA was detected by RT-PCR. Total RNA was isolated from the cells with TRIzol reagent (Promega). The cDNA was synthesized using VP1-P2: 5′- GCT CTA GAT TAC TGT TTC TCA GGT GCA -3′ as primer and Moloney Murine Leukemia Virus reverse transcriptase (Promega) in the reverse transcription reaction. Amplification was performed using the following primer pairs: VP2-P1 and VP2-P2 for VP2, VP3-P1 and VP3-P2 for VP3, VP4-P1 and VP4-P2 for VP4, β-actin-P1: 5′- GAT ATG GAG AAG ATC TGG CA-3′ and β-actin-P2: 5′- GTT GAA TGT AGT TTC GTG GA-3′ for β-actin. Amplified cDNA was analyzed with 1% agarose gel electrophoresis.

### Preparation of transgenic donor cells

Donor cell lines were established from a 50-days fetus of Holstein cow as described previously [33]. In briefly, a pregnant cow was detected and confirmed by transrectal ultrasound at embryonic days 50, after anesthesia, the fetus was obtained with surgery from pregnant cow. The fetus was sacrificed by decapitation, and fetal tissue was minced, suspended in DMEM/Ham's F12 (1∶1) supplemented with 15% FBS and antibiotics, seeded in 25 cm^2^ tissue culture flasks, and cultured at 37°C in a humidified atmosphere of 5% CO_2_ in air for several days. 10 µg of shRNA recombinant lentivirus vector was transfected into bovine fetal fibroblast cells using Lipofectamine 2000 according to manufacturer's instruction (Invitrogen). After 48 h, the cells were subcultured. 14 days later, transgenic bovine fetal fibroblast cells were screened by FACS, and confirmed by PCR with primer pair LT1/LT2 and DNA sequencing.

### Oocyte in vitro maturation

Oocytes were maturated in vitro as described previously [33]. Bovine ovaries were collected from the Jinan Bovine abattoir and cumulus-enclosed oocyte complexes were aspirated from 3 to 8 mm follicles. Oocytes with evenly shaded cytoplasm and intact layers of cumulus cells were selected and cultured in maturation medium: M199 containing 10% fetal bovine serum (FBS; HyClone Laboratories, Logan, UT), 0.5 µg/ml FSH (Sioux Biochemicals, Sioux City, IA), 5 µg/ml LH (Sioux Biochemicals), 100 U/ml penicillin and 100 µg/ml streptomycin (HyClone Laboratories, Logan, UT) and cultured for 20 h prior to nuclear transfer.

### Nuclear transfer

Routine manipulations were previously described [33]. After maturation for 20 h, the cumulus cells were removed and oocytes with the first polar body were enucleated as cytoplast recipients. Single transfected cells were transferred to the perivitelline space of enucleated oocytes. The reconstructed couplets were fused in mannitol fusion buffer by two DC pulses of 1.8 kV/cm for 20 µs. Then the fused clones were activated by 5 µM ionomycin for 5 min and treated with 10 mg/ml cycloheximide in CR1aa medium for 5 h in 5% CO_2_ in air at 38°C. Following activation, the embryos were cultured in CR1aa medium supplemented with BSA for 40 h, then the cleaved embryos were transferred and cultured in CR1aa medium plus 4% FBS feeding with a single layer cumulus cells under 5% CO_2_ in air at 38°C with high humidity.

### Embryo transfer

On day 6 or 7, compacted morulae and blastocysts were shipped overnight in equilibrated CR2 at 38.5°C to the site of transfer. One to two embryos were transferred nonsurgically to cows synchronized 1 day to the stage of embryonic development. Pregnancy was detected by transrectal ultrasound at embryonic days 60, and pregnant recipients were checked by ultrasound or palpation at approximately 30-days intervals to confirm ongoing pregnancies, and fetuses were obtained with surgery after anesthesia from pregnant cows at four months.

### shRNA integration into chromosome of cloned bovine fetuses

The genomic DNA from the ear tissue of 4-month-old cloned and normal fetuses were extracted according to standard molecule cloning instructions, and used as the template for PCR amplification with LT1/LT2 primer pair, then shRNA transgenic fragment was confirmed by DNA sequencing.

Up and Down primers of probe for Southern Blotting were designed according to the flanking sequence of shRNA in shRNA recombinant H1 Lenti-virus vector, Up primer 1: 5′- GAA GAA CGG CAT CAA GGT G -3′; Down primer: 5′- TTG CTT CCC GTA TGG CTT -3′; The shRNA recombinant H1 Lenti-virus vector was used for template to amplify 414 bp probe labeling with Invitrogen Platinum® Tag Kit. The genomic DNAs from the cloned and normal fetus were digested into smaller fragments with excess amounts of *Xho*I and *Apa*I for 18 h. The concentrated samples were hybridized with probe as standard protocol.

### shRNA expression in primary bovine tongue epithelium cells

Total RNA was isolated from the cells with TRIzol reagent (Promega). Ten µg of total RNA were then size fragmented by electrophoresis on 1.5% agarose/formaldehyde gel, transferred onto Nitrocellulose membrane, prehybridized, and hybridized according to the method of Church and Gilbert [34]. The cDNA was prepared with SuperScript. III First-Strand Synthesis System (invitrogen) from the total RNA using oligo dT primer. Up primer of bovine β-actin: 5′- AGC AAG CAG GAG TAC GAT GAG -3′, Down primer of bovine β-actin: 5′- TGC CAA TCT CAT CTC GTT TTC -3′, and cDNA was used for template to amplify 313 bp of β-actin fragment. The shRNA probe was artificially synthesized as follows, sense chain: 5′- CCA GTC AGG CAA CAC TGG A -3′, anti-sense chain: 5′- TCC AGT GTT GCC TGA CTG G -3′, and then was annealing to double strain probe of shRNA. The complementary DNA probes were labeled with rediprimer™ II random primer labeling system (amersham) to yield a specific activity of 2–5×10^8^ cpm/µg, and used at a concentration of 10^6^ cpm/ml hybridization solution. The final posthybridization wash was performed four times at 65°C for 10 min each time with a solution containing 1% SDS, 1 mM EDTA, and 20 mM sodium phosphate, pH 7.2. The membranes were then exposed to X-ray film (Kodak) with intensifying screens at −80°C. For the reprobing, the original probe was removed by treating the membranes with boiling water for 10 min.

### FMDV infection in primary bovine tongue epithelium cells

The primary normal or transgenic bovine tongue cells cultured in 96-well plates were inoculated with 100 TCID_50_ of the FMDV strain each well, after 1 h absorption, the medium was changed with fresh medium without FCS, and virus samples were collected at designated time points and frozen at −80°C until for assessment of FMDV titer and viral RNA, respectively.

The viral RNA was isolated from virus sample using a TRIzol kit (Invitrogen), and cDNA was prepared with SuperScript. III First-Strand Synthesis System (invitrogen) and RT primers for viral RNA as follows, R1 5′- CTC TGT AGT CAC TGT CTG TC -3′, and oligo dT for bovine β-actin. The cDNA then was subjected to real-time RT-PCR analysis, according to the manufacturer's instruction of QuantiTect SYBR green RT–PCR kit (Qiagen). Cycling program was set as following: 95°C for 10 min for the PCR initial activation and 40 cycles of denaturation at 95°C for 15 s, annealing and extension at 60°C for 1 min. PCR primer pair were used as follows, R1 and F1: 5′-CAA AAG ATG GTC ATG GGC-3′ for viral RNA. The standard used was 10-fold dilutions of 10^7^ TCID_50_ of virus ml^−1^. All further computations were done using ABI Prism 7000 SDS Software (Applied Biosystems). The statistical analysis was based on data from three independent experiments.
